# Development of machine learning models to predict RT-PCR results for severe acute respiratory syndrome coronavirus 2 (SARS-CoV-2) in patients with influenza-like symptoms using only basic clinical data

**DOI:** 10.1186/s13049-020-00808-8

**Published:** 2020-12-01

**Authors:** Thomas Langer, Martina Favarato, Riccardo Giudici, Gabriele Bassi, Roberta Garberi, Fabiana Villa, Hedwige Gay, Anna Zeduri, Sara Bragagnolo, Alberto Molteni, Andrea Beretta, Matteo Corradin, Mauro Moreno, Chiara Vismara, Carlo Federico Perno, Massimo Buscema, Enzo Grossi, Roberto Fumagalli

**Affiliations:** 1 Department of Medicine and Surgery, University of Milan-Bicocca, Monza, Italy; 2 Department of Anaesthesia and Intensive Care Medicine, Niguarda Ca’ Granda, Milan, Italy; 3 Department of General oncologic and mini-invasive Surgery, Niguarda Ca’Granda, Milan, Italy; 4 Department of Emergency Medicine, Niguarda Ca’ Granda, Milan, Italy; 5 Medical Department, Niguarda Ca’ Granda, Milan, Italy; 6 Department of Laboratory Medicine, ASST Niguarda Hospital, University of Milan, Milan, Italy; 7 Semeion Research Center of Sciences of Communication, Rome, Italy; 8 Department of Mathematical and Statistical Sciences, University of Colorado at Denver, Denver, CO USA; 9 Centro Diagnostico Italiano, Milan, Italy; 10 Villa Santa Maria Foundation, Tavernerio, Italy

**Keywords:** Severe acute respiratory syndrome coronavirus 2, Emergency service, hospital, Pandemics, Critical care, Artificial intelligence, Supervised machine learning

## Abstract

**Background:**

Reverse Transcription-Polymerase Chain Reaction (RT-PCR) for Severe Acute Respiratory Syndrome Coronavirus 2 (SARS-COV-2) diagnosis currently requires quite a long time span. A quicker and more efficient diagnostic tool in emergency departments could improve management during this global crisis. Our main goal was assessing the accuracy of artificial intelligence in predicting the results of RT-PCR for SARS-COV-2, using basic information at hand in all emergency departments.

**Methods:**

This is a retrospective study carried out between February 22, 2020 and March 16, 2020 in one of the main hospitals in Milan, Italy. We screened for eligibility all patients admitted with influenza-like symptoms tested for SARS-COV-2. Patients under 12 years old and patients in whom the leukocyte formula was not performed in the ED were excluded. Input data through artificial intelligence were made up of a combination of clinical, radiological and routine laboratory data upon hospital admission. Different Machine Learning algorithms available on WEKA data mining software and on Semeion Research Centre depository were trained using both the Training and Testing and the K-fold cross-validation protocol.

**Results:**

Among 199 patients subject to study (median [interquartile range] age 65 [46–78] years; 127 [63.8%] men), 124 [62.3%] resulted positive to SARS-COV-2. The best Machine Learning System reached an accuracy of 91.4% with 94.1% sensitivity and 88.7% specificity.

**Conclusion:**

Our study suggests that properly trained artificial intelligence algorithms may be able to predict correct results in RT-PCR for SARS-COV-2, using basic clinical data. If confirmed, on a larger-scale study, this approach could have important clinical and organizational implications.

**Supplementary Information:**

The online version contains supplementary material available at 10.1186/s13049-020-00808-8.

## Background

At the end of 2019, an outbreak of pneumonia, of unknown origin at the time, turned out to have stemmed from Wuhan, Hubei, China and consequently spread throughout the world, reaching Italy in February 2020 [1–3].

A new betacoronavirus, subsequently named Severe Acute Respiratory Syndrome Coronavirus 2 (SARS-COV-2), was identified as the cause of the epidemic [4]. The infection with SARS-COV-2 has, in humans, a broad spectrum of clinical presentations [5] and was named Coronavirus Disease 2019 (COVID-19). Initial symptoms are nonspecific and similar to other seasonal viral diseases, which entail fever, dry cough and fatigue. This could lead to a fairly impossible clinical diagnosis. Therefore, etiological diagnosis relies on Reverse Transcription-Polymerase Chain Reaction (RT-PCR) to detect the genome of SARS-COV-2.

There are several important limitations to RT-PCR. First, current techniques take up to 6–8 h in order to obtain plausible results and often laboratories cannot handle the overload. Second, not all hospitals have the equipment and staff to run tests 24/7. Consequently, swabs are sent out to other facilities, thus slowing down the process and swamping central laboratories. Third, RT-PCR, on a retropharyngeal swab, may result falsely negative in the initial phase of the disease, in spite of the presence of typical symptoms [6–8]. Last of all, this technique carries a certain cost, which could mean a considerable financial burden weighing upon both health systems and patients.

A direct consequence of these limitations is the time spent by a large number of patients awaiting results in the emergency department before a decision can be taken as to where admit them to, e.g. in wards and intensive care units focused on COVID-19 patients, or in “non-infective” wards of the hospital [2, 9–12]. This is particularly troublesome for critically ill patients requiring immediate endotracheal intubation and mechanical ventilation. Specialized medical staff need to attend to these patients in emergency departments where, however, the health system is already under a lot of pressure.

Finding an easy and fast method of predicting positivity or negativity to SARS-COV-2, would prove to be of great clinical value. Algorithms have already been proposed using advanced imaging, e.g. chest computed tomography (CT) [13, 14]. However, not all hospitals or countries can carry out a CT scan on every patient.

The main goal of our study was therefore to develop a machine learning model to predict the results of RT-PCR for SARS-COV-2 using only basic clinical, radiological and routine laboratory data at hand in all emergency departments.

We hypothesized that using Artificial Neural Networks (ANNs), and other Machine Learning Systems (MLS), would allow to obtain accurate results on RT-PCR testing for SARS-COV-2 and that these systems could possibly be applied in the future. The performance of different ANNs and MLS was analyzed so as to distinguish between patients resulting positive or negative to SARS-COV-2, thus identifying those variables which express the maximum amount of relevant information.

## Methods

### Study design and selection of participants

This retrospective, single centre study, was approved by the Institutional Review Board of our hospital (N°3733). The need for informed consent from individual patients was waived owing to the retrospective nature of the study. While not developed specifically for models using machine learning [15], the study followed the guidelines of the Transparent reporting of a multivariable prediction model for individual prognosis or diagnosis (TRIPOD) [16].”

All patients admitted to the emergency department of our hospital between February 22, 2020 and March 16, 2020, were screened for eligibility. Symptoms of presentation compatible with COVID-19 (fever, sore throat, cough, dyspnoea, chest pain, headache, syncope, asthenia, arthralgia, diarrhoea, nausea and vomit) constituted the inclusion criteria. Age < 12 years and absence of evaluation of the leukocyte formula (defined as percentages of the five types of leukocytes: neutrophils, lymphocytes, eosinophils, basophils and monocytes) in the emergency department constituted exclusion criteria.

### Data collection

Clinical data regarding the admission to the emergency department were retrieved from the Patient Data Management System of our hospital. Data included age, gender, presence and type of comorbidities, reported symptoms and medication currently being taken. Each drug was placed in a specific category.

In our hospital, there is no formal checklist to collect the medical and medication history. Therefore, if specified by the ER physicians, comorbidities and medication use were considered as present, otherwise if not specified, they were considered as absent. In addition, information regarding vital signs upon admission to the emergency department (first measurement), presence and type of ventilatory support, routinely performed blood tests, major electrocardiographic characteristics (presence of sinus rhythm and ST abnormalities) and chest X-rays (presence of any type of parenchymal involvement, presence of pleural effusion) were collected.

The results of the RT-PCR swab for SARS-COV-2 were recorded. Should the outcome have proved negative in a symptomatic patient, our hospital made it mandatory for a second swab to be carried out after 48 h and these swabs were checked to confirm negativity. The complete list of collected variables is summed up in Table 1.

**Table 1:** Variables under study (*n* = 74)

**Demographic Data**	
Age (years)	
Sex (M; F)	
**Medical History (yes; no)**	
Asthma	
Atrial fibrillation	
Autoimmune/inflammatory disease	
Chronic Interstitial lung disease	
Chronic kidney disease	
Chronic liver disease	
Chronic obstructive pulmonary disease (COPD)	
Diabetes mellitus	
Hematologic malignancy	
Human immunodeficiency virus	
Hypertension	
Ischemic cardiomyopathy	
Smoking (active)	
Solid organ tumor	
**Medication history (yes; no)**	
5-alpha-reductase inhibitors	
Angiotensin converting enzyme inhibitor	
Anti-arrhythmic drugs	
Antibiotics before Emergency Department	
Anticoagulant therapy	
Anti-epileptic therapy	
Anti-platelets therapy	
Beta blocker	
Calcium channel antagonist	
Diuretics	
Drugs for psychiatric disorders	
Hydroxymethylglutaryl-CoA reductase inhibitors	
Immunosuppressant drugs	
Sartans	
**Reported Signs and Symptoms (yes; no)**	
Arthralgia	
Asthenia	
Chest pain	
Cough	
Dyspnea	
Fever	
Gastrointestinal symptoms	
Headache	
Sore throat	
Syncope	
**Vital signs, ECG and Chest X-ray findings**	
Glasgow coma scale, score	
Body temperature, Celsius	
Systolic blood pressure, mmHg	
Diastolic blood pressure, mmHg	
Heart rate, beats per min	
Sinus rhythm – no. (%)	
Normal ST segment – no. (%)	
Respiratory rate, breaths per min	
Oxygen supplementation – no. (%)	
Non-Invasive Ventilation – no. (%)	
Chest X-rays opacity – no. (%)	
Chest X-rays pleural effusion – no. (%)	
**Laboratory findings**	
Total white blood cell count, 10^3^/μL	
Neutrophils, %	
Lymphocytes, %	
Monocytes, %	
Eosinophils, %	
Basophils, %	
Total red blood cells count, 10^6^/μL	
Haematocrit, %	
Haemoglobin, g/dL	
Mean corpuscular haemoglobin concentration, g/dL	
Mean corpuscular haemoglobin, pg	
Mean red blood cell volume, μm^3^	
Red blood cells distribution width,**%**	
Platelets, 10^3^/μL	
Glycaemia, mg/dL	
Creatinine, mg/dL	
Urea, mg/dL	
Sodium, mEq/L	
Total bilirubin, mg/dL	
C-reactive protein, mg/dL	
**Swab test for SARS-COV-2 (TARGET)**	
Negative (yes; no)	
Positive (yes; no)	

Medical and medication history and reported sign and symptoms were collected by physicians in the Emergency Room. Clinical findings refer to data gathered in the Emergency Room at hospital admission

### Statistical analysis and sample size

All data were tested for consistency with variance and normality of distribution using the Shapiro-Wilk test. Normally distributed data were expressed as mean ± standard deviation, while non-normally distributed data were reported as median and interquartile range. Binary data were summed up in percentages, frequency of occurrence, and compared through Chi-Square test. Continuous variables were compared through Student T-Test or Wilcoxon Rank-Sum test, as appropriate.

Pearson’s and Spearman’s correlation was used to assess the correlation between collected variables (continuous and nominal, respectively) and RT-PCR for SARS-COV-2 results. A *P*-value lower than 0.05 was considered as statistically significant. Analysis was performed with SigmaPlot v.12.0 (Systat Software Inc., USA).

The study was carried out on a convenience sample of 200 patients admitted to the emergency department and tested for SARS-COV-2.

### Machine learning methods

#### Training and testing validation protocol

The target variable to be predicted was the result of the RT-PCR for SARS-COV-2 performed in the emergency department. In order to predict and estimate the results of the RT-PCR for SARS-COV-2 using an input of 74 variables under study (Table 1), different Machine Learning algorithms available on WEKA data mining software [17–19] and on Semeion Research Centre depository (Massimo Buscema, Deep Supervised ANNs, Semeion SW #12, version 33.0, 1999–2019) were trained. These classification tools were first applied to predict RT-PCR results using the Training and Testing validation protocol, with the following steps:

1. Subdivision of the dataset into two sub-samples, A and B, each containing 50% of records and having an equal proportion of cases and controls (in our case SARS-COV-2 positive and SARS-COV-2 negative). The two sub-samples were obtained through the application of the TWIST algorithm, (i.e. they were not obtained by random extraction), in order to create two subsamples with similar probability density for all the input variables (see below). A homogeneity check was performed to confirm the substantial equivalence of the two subsets with regard to the distribution of variables. In the first run, A was used as Training Set and B as Testing Set.
2. Application of ANN on the Training Set. In this phase, the ANN learned to associate the input variables with those indicated as targets.
3. After the training phase, the weights matrix produced by the algorithm was saved and frozen together with all parameters used for the training.
4. The Testing Set was then shown to a virgin twin (same architecture and base parameters) ANN with the same weights matrix of the trained ANN, acting as final classifier. This operation took place for all records and the results (right or wrong classification) was not communicated to the classifier. This allowed to assess the generalization ability of trained ANN.
5. In a second run, another virgin ANN was applied to subset B which was used as training subset and then to subset A which was used as testing subset.
6. Therefore, the results are relevant to two sequences of training testing protocol: A-B and B-A.

Results were drawn up in terms of sensitivity (correct classification of positive patients), specificity (correct classification of negative patients), global accuracy (arithmetic mean between sensitivity and specificity). Overall results are expressed as average of the two experiments. This crossover procedure allows to classify blindly all records with the trained algorithm ensuring the generalization capability of the model on records never seen before.

The machine learning algorithms developed at the University of Waikato, New Zealand, available on the WEKA data mining software are listed in Table 2 [20–26], while two ANNs (Self Momentum Back Propagation and Sine Net) [29, 30] were implemented in “Supervised ANNs Software”, developed at the Semeion Research Center in Rome, Italy (Buscema; Supervised ANNs. Semeion software #12, version 33.0). Table 3 shows the main features of Semeion Machine Learning Systems.

**Table 2:** Nicknames of Machine Learning Systems in WEKA software Package

Machine Learning software	Nick name
Logistic [20]	Logistic
Pruned decision tree [21]	J 48
Multi Layer Perceptron [22]	MLP
NaivBayes [23]	NaivBayes
RandomForest [24]	RandomForest
RotationForest [25]	RotationForest
Sequential Minimal Optimization [26]	SMO
K Nearest Neighborhood [27]	KNN47
Additive Logistic Regression [28]	Logit Boost48

**Table 3:** Main Features of Semeion Machine Learning Systems employed in the study

ANNs Architecture	Hidden layers	Learning Rate	Epochs	Initialization	Output Function	Nickname
Conic Net [31]	5 Layers= 4x12x12x12	0.01	1000	Auto-encoders	Soft Max	D_FF_Conic (4x12x12x12)
	5 Layers= 6x12x12x12	0.01	1000	Auto-encoders	Soft Max	D_FF_Conic (6x12x12x12)
Sine Net [32–34]	1 Layer = 48	0.1	2000	Random	Soft Max	D_FF_Sn [35]
Back Propagation [29]	0 Layer = L	0.1	1000	Random	Soft Max	D_FF_Bp (0)
	1 Layer = 24	0.1	1000	Random	Soft Max	D_FF_Bp [22]
	5 Layers= 16x16x16x16	0.01	2000	Auto-encoders	Soft Max	D_FF_Bp (16x16x16x16)
Bi-Modal Net	1 Layer = 48	0.1	1000	Random	Soft Max	D_FF_Bm [35]
Gauss Net [36]	1 Layer = 64	0.01	1000	Random	Soft Max	D_FF_GNet [37]

However, since noisy input attributes can sometimes hide the small meaningful information embedded in other attributes, a pruning procedure was used as a pre-processing tool to eliminate noisy variables before the outcome prediction of the main test. In order to conduct this procedure, an input selection algorithm named TWIST (Training With Input Selection and Testing) was applied [38, 39]. This selection algorithm was recently developed at the Semeion Research Center in Rome, Italy (Buscema M (2006–2012) TWIST Input Search, Semeion software #39, version 5.1, 2006–2016).

#### TWIST algorithm

As previously shown [40], the TWIST algorithm is a complex algorithm able to search for the best distribution of the global dataset divided in two optimally balanced subsets containing a minimum number of input features, useful for optimal pattern recognition. TWIST is an evolutionary algorithm based on a paper about Genetic Doping Systems [19], which has already been applied to medical data with very promising results [40–44]. A detailed description of the algorithm is available in the Online supplement.

#### 5-K-fold

In addition to the Training and Testing validation protocol, a 5 K-fold cross-validation protocol was applied [45], in order to analyze data also with a standard and popular approach. The dataset was randomly split in 5 groups (folds) with a similar number of subjects. Each unique group was used as a hold out, or validation dataset, and the remaining groups were used as training datasets. The model fitted on the training set was evaluated on the validation set. Five different models for each employed machine learning system were created, and each model provided an evaluation score. The skill of the 5 models was summarized as mean sensitivity, specificity, overall accuracy and balanced accuracy.

#### Assessment of model calibration

Cost Curves [46] were applied to the studied algorithms in order to assess model calibration (Results presented in the Online Supplement).

## Results

Three hundred forty-seven patients fulfilled the inclusion criteria, 148 patients presented exclusion criteria (9 patients < 12 years, 139 patients without leukocyte formula), leaving 199 patients for the analysis.

### Population description and classic statistics

Table 4 summarizes the main characteristics of the overall study population (*n* = 199) and of the two subgroups, i.e. patients who tested positive (*n* = 124) and negative (*n* = 75) to SARS-COV-2. There were no missing data regarding the recorded variables. A comparison between the characteristics of the study population (n = 199) and of patients excluded due to the absence of leukocyte formula (*n* = 139) can be found in the Online Data Supplement (Table S3).

**Table 4:** Characteristics of the study population

Demographic characteristics	Overall population (***n*** = 199)	Positive (***n*** = 124)	Negative (***n*** = 75)	***P***
Age, years	65 [46–78]	65 [49–77]	66 [38–82]	0.94
Male – no. (%)	127 (63.8)	78 (62.9)	49 (65.3)	0.85
**Medical History – no. (%)**				
Asthma	13 (6.6)	9 (7.3)	4 (5.4)	0.83
Atrial fibrillation	19 (9.6)	11 (8.9)	8 (10.7)	0.87
Autoimmune/inflammatory disease	6 (3.0)	3 (2.4)	3 (4)	0.84
Chronic Interstitial lung disease	1 (0.5)	1 (0.8)	0 (0.0)	0.80
Chronic kidney disease	11 (5.5)	8 (6.5)	3 (4.0)	0.68
Chronic liver disease	7 (3.5)	3 (2.4)	4 (5.3)	0.49
Chronic obstructive pulmonary disease	15 (7.5)	5 (4.0)	10 (13.3)	0.03
Diabetes mellitus	29 (14.6)	16 (12.9)	13 (17.3)	0.51
Hematologic malignancy	6 (3.0)	4 (3.2)	2 (2.7)	0.84
Human immunodeficiency virus	1 (0.5)	0 (0.0)	1 (1.3)	0.80
Hypertension	85 (42.7)	55 (44.4)	30 (40)	0.65
Ischemic cardiomyopathy	19 (9.6)	10 (8.1)	9 (12.0)	0.51
Smoking (active)	12 (6.0)	4 (3.2)	8 (10.7)	0.07
Solid organ tumour	16 (8.0)	7 (5.7)	9 (12.0)	0.18
**Medication history – no. (%)**				
5-alpha-reductase inhibitors	12 (6.0)	9 (7.3)	3 (4.0)	0.53
Angiotensin converting enzyme inhibitors	33 (16.6)	21 (16.9)	12 (16.0)	0.98
Anti-arrhythmic drugs	11 (5.5)	6 (4.8)	5 (6.7)	0.82
Antibiotics before Emergency Department	47 (23.6)	36 (29.0)	11 (14.7)	0.03
Anticoagulant drugs	25 (12.6)	15 (12.1)	10 (13.3)	0.97
Anti-epileptic drugs	10 (5.0)	1 (0.8)	9 (12.0)	0.002
Antiplatelets drugs	33 (16.6)	20 (16.1)	13 (17.3)	0.98
Beta blockers	40 (20.1)	24 (19.4)	16 (21.3)	0.88
Calcium channel antagonists	27 (13.6)	18 (14.5)	9 (12.0)	0.77
Diuretics	35 (17.6)	21 (16.9)	14 (18.7)	0.91
Drugs for psychiatric disorders	7 (3.5)	1 (0.8)	6 (8.0)	0.02
Hydroxymethylglutaryl-CoA (HMG-CoA) reductase inhibitors	28 (14.1)	18 (14.5)	10 (13.3)	0.98
Immunosuppressant drugs	12 (6.0)	8 (6.5)	4 (5.3)	0.99
Sartans	24 (12.1)	19 (15.3)	5 (6.7)	0.11
**Reported Signs and Symptoms - no. (%)**				
Arthralgia	7 (3.5)	6 (4.8)	1 (1.3)	0.37
Asthenia	18 (9.1)	9 (7.3)	9 (12.0)	0.38
Chest pain	7 (3.5)	7 (5.7)	0 (0.0)	0.09
Cough	130 (65.3)	91 (73.4)	39 (52)	0.004
Dyspnea	75 (37.7)	43 (34.7)	32 (42.7)	0.33
Fever	174 (87.4)	119 (96.0)	55 (73.3)	< 0.001
Gastrointestinal symptoms	27 (13.6)	18 (14.5)	9 (12.0)	0.77
Headache	12 (6.0)	8 (6.5)	4 (5.3)	0.99
Sore throat	8 (4.0)	3 (2.4)	5 (6.7)	0.27
Syncope	5 (2.5)	2 (1.6)	3 (4.0)	0.57
**Vital signs, ECG and Chest X-ray findings**				
Glasgow coma scale, score	15 [15–15]	15 [15–15]	15 [15–15]	> 0.99
Body temperature, Celsius	37.6 ± 0.9	37.8 ± 0.8	37.2 ± 1	< 0.001
Systolic blood pressure, mmHg	131 ± 22	133 ± 19	128 ± 25	0.09
Diastolic blood pressure, mmHg	75 [65–80]	75 [70–80]	70 [65–80]	0.09
Heart rate, beats per min	90 [80–105]	90 [83–105]	90 [80–105]	0.87
Sinus rhythm – no. (%)	185 (93)	117 (94.4)	68 (90.7)	0.48
Normal ST segment – no. (%)	198 (99.5)	123 (99.2)	75 (100.0)	0.80
Respiratory rate, breaths per min	18 [16–22]	18 [16–22]	18 [16–24]	0.80
Oxygen supplementation – no. (%)	51 (25.6)	27 (21.8)	24 (32.0)	0.15
Non-Invasive Ventilation – no. (%)	8 (4.0)	4 (3.2)	4 (5.3)	0.72
Chest X-rays opacity – no. (%)	158 (79.4)	104 (83.9)	54 (72.0)	0.07
Chest X-rays pleural effusion – no. (%)	20 (10.1)	7 (5.7)	13 (17.3)	0.01
**Laboratory findings**				
Total white blood cell count, 10^3^/μL	6.64 [4.65–9.65]	5.44 [4.21–7.23]	9.28 [6.87–13.64]	< 0.001
Neutrophils, %	72.5 [64.4–81.7]	70.4 [62.5–79.9]	76.7 [68.4–85.3]	0.001
Lymphocytes, %	18.0 [10.5–25.1]	20.6 [12.9–27.7]	14.5 [7.4–19.8]	< 0.001
Monocytes, %	7.9 [5.6–10.2]	8.0 [6–10.9]	7.6 [5.4–9.7]	0.18
Eosinophils, %	0 [0–0.3]	0 [0–0.2]	0.2 [0–1.2]	< 0.001
Basophils, %	0.3 [0.2–0.4]	0.2 [0.2–0.4]	0.3 [0.2–0.5]	0.03
Total red blood cells count, 10^6^/μL	4.80 [4.29–5.25]	4.88 [4.42–5.28]	4.52 [4.01–5.14]	0.01
Haematocrit, %	41.6 ± 5.3	42.1 ± 4.5	40.8 ± 6.3	0.10
Haemoglobin, g/dL	13.7 ± 1.9	14.0 ± 1.6	13.3 ± 2.2	0.01
Mean corpuscular haemoglobin concentration, g/dL	33.1 [32–33.9]	33.2 [32.5–34.1]	32.8 [31.6–33.8]	0.01
Mean corpuscular haemoglobin, pg	29.2 [28.1–30.5]	29.2 [27.9–30.5]	29.3 [28.6–30.2]	0.56
Mean red blood cell volume, μm^3^	88.3 [85.4–91.4]	87.7 [84.5–90.4]	89.7 [87–92.6]	0.002
Red blood cells distribution width,**%**	13.2 [12.4–14.4]	13.1 [12.3–13.9]	13.6 [12.5–15.5]	0.01
Platelets, 10^3^/μL	193 [162–244]	183 [145–234]	221 [175–283]	< 0.001
Glycaemia, mg/dL	120 [104–142]	120 [104–138]	117 [101–158]	0.80
Creatinine, mg/dL	1.0 [0.8–1.3]	0.98 [0.82–1.21]	1.09 [0.83–1.45]	0.19
Urea, mg/dL	35 [23–54.5]	34 [22–49]	41.5 [26–64.5]	0.07
Sodium, mEq/L	138 [135–140]	138 [134–140]	138 [136–141]	0.37
Total bilirubin, mg/dL	0.5 [0.4–0.7]	0.5 [0.3–0.6]	0.5 [0.4–0.7]	0.05
C-reactive protein, mg/dL	4.1 [1.2–9]	4.0 [1.3–8.9]	4.5 [0.9–9.3]	0.90

Variables are ordered by categories. Continuous variables are expressed as median [interquartile range] or mean ± Standard Deviation, as appropriate. Medical and medication history, reported signs and symptoms were collected from physicians of the Emergency Department. Clinical findings (Vital signs, ECG – Electrocardiogram, Chest X-ray and Laboratory findings) refer to the first measurements performed in the Emergency department

Median age in the overall population was 65 [46–78] years, with similar distribution in the two subgroups (65 [49–77] vs. 66 [38–82] years *p* = .94, for positive and negative patients, respectively). Most patients were male (63.8%) with no significant difference between the two subgroups (62.9% vs. 65.3%, *p* = .85). The most common comorbidities were hypertension (42.7%) and diabetes (14.6%) with similar prevalence between patients with and without SARS-COV-2. A lower prevalence of Chronic obstructive pulmonary disease (COPD) was observed in patients with COVID-19 (4.0% vs. 13.3% *p* = .03).

Regarding current medications, positive patients had lower chronic use of anti-epileptics and drugs for psychiatric disorders, as compared to patients that tested negative (0.8% vs. 12.0%, *p* = .002 and 0.8% vs. 8.0%, *p* = .02, respectively). Furthermore, administration of antibiotics before access to the emergency department was higher in the positive subgroups (29.0% vs. 14.7%, p = .03).

As for reported signs and symptoms, fever and coughing were more prevalent in patients with COVID-19 (96.0% vs. 73.3%, *p* < .001 and 73.4% vs. 52.0%, *p* = .004, respectively).

Several clinical findings turned out to be significantly different between the two population studies (Table 4). In particular, SARS-COV-2 positive patients had a slightly higher external body temperature (37.8 ± 0.8 vs. 37.2 ± 1.0, p = <.001), lower prevalence of pleural effusion at chest X-ray (5.7% vs. 17.3%, *p* = .01) and a significant difference regarding the complete blood count and leukocyte formula (Table 4).

Table 5 summarizes the positive and negative linear correlation index between descriptive variables and the results of RT-PCR for SARS-COV-2.

**Table 5:** Study variables positively and negatively correlated with SARS-COV-2 positivity

**Positive linear correlation**			
**VARIABLES**	**Pearson R**	**VARIABLES**	**Pearson R**
*Fever	0.33	*Sinus Rhythm	0.06
*Body temperature (degree Celsius)	0.27	*Chronic kidney disease	0.05
Lymphocytes percentage	0.26	*Monocytes percentage	0.05
Cough	0.22	Hypertension	0.04
*Total red blood cells count (10^6^/μL)	0.19	Asthma	0.04
*Haemoglobin (g/dL)	0.17	*Age	0.04
Glasgow coma scale (GCS)	0.17	Gastrointestinal symptoms	0.04
*Antibiotics before Emergency Department	0.16	*Calcium channel antagonist	0.04
*Chest pain	0.15	Mean corpuscular haemoglobin concentration (%)	0.03
*Chest X-rays opacity	0.14	Respiratory rate (breaths/min)	0.03
*Sartans	0.13	*Female	0.02
*Systolic blood pressure (mmHg)	0.12	*Headache	0.02
Haematocrit (%)	0.12	*Immunosuppressant drugs	0.02
Arthralgia	0.09	*Hydroxymethylglutaryl-CoA (HMG-CoA) reductase inhibitors	0.02
*Diastolic blood pressure (mmHg)	0.07	Hematologic malignancy	0.02
P wave present and normal	0.07	*Angiotensin converting enzyme inhibitor	0.01
*5-alpha-reductase inhibitors	0.07	Heart rate (beats per minute)	0.00
Chronic Interstitial lung disease	0.06		
**Negative linear correlation**			
**VARIABLES**	**Pearson R**	**VARIABLES**	**Pearson R**
*Total white blood cells count (10^3^/μL)	−0.46	*Dyspnoea	−0.07
*Eosinophils percentages	−0.34	*Ischemic cardiomyopathy	−0.06
Platelets (10^3^/μL)	− 0.33	*Basophils percentage	− 0.06
*Anti-epileptic therapy	−0.25	Diabetes mellitus	−0.06
*Calcium (mg/dL)	−0.22	Creatinine (mg/dL)	−0.06
Mean red blood cell volume (μm^3^)	−0.21	*Mean corpuscular haemoglobin (pg)	−0.06
Neutrophils percentages	−0.19	*Non Invasive Ventilation	−0.05
*Drugs for psychiatric disorders	−0.19	Autoimmune/inflammatory disease	−0.04
*Chest X-rays pleural effusion	−0.19	Red blood cells distribution width (%)	−0.04
*Chronic obstructive pulmonary disease	−0.17	Anti-arrhythmic drugs	−0.04
*Smoking (active)	−0.15	Syncope	−0.04
*Urea (mg/dL)	−0.14	Glycaemia (mg/dL)	−0.03
*Total bilirubin (mg/dL)	−0.14	*Diuretics	−0.03
Oxygen supplementation	−0.11	Atrial fibrillation	−0.03
*Solid organ tumour	−0.11	C-reactive protein (mg/dL)	−0.03
*Sore throat	−0.10	*Male	−0.02
Human Immunodeficiency Virus	−0.09	Beta blocker	−0.02
Asthenia	−0.08	Anticoagulant therapy	−0.02
Chronic liver disease	−0.08	*Anti-platelets therapy	−0.02
*Sodium (mEq/L)	−0.07		

Variables were divided according to their positive or negative correlation to the target variable (positivity of RT-PCR for SARS-COV-2) and are listed in decreasing order of positive or negative correlation coefficient, respectively. Asterisks indicate variables included in the TWIST model

### Prediction of the RT-PCR outcome with machine learning algorithms

The TWIST system selected 42 variables of the original attributes. Selected variables are marked with and asterisk in Table 5. A global dataset of 42 input and 2 target attributes was thus generated. Thereafter, two optimal subsets were created, in order to apply the training and testing procedure described above.

Table 6 shows the results obtained by the application of an array of machine learning systems to the variables selected by TWIST system. These results are the average of two training-testing procedures (A-B and B-A sequences). Detailed predictive results for each experiment (A-B and B-A) is available in Table S1 in the Online Supplement.

**Table 6:** Predictive results with variables selection using Semeion (^a^) and WEKA (^b^) Machine learning systems

Machine learning system	Sensitivity	Specificity	Overall accuracy	Balanced accuracy	Variance	PPV	AUROC
**D_FF_Conic(4x12x12x12)**^a^	94.1	88.7	91.4	92.2	0.5	93.5	0.90
**D_FF_Conic(6x12x12x12)**^a^	92.5	90.2	91.3	91.7	0.0	94.1	0.91
**D_FF_Bp(24)** ^a^	89.2	93.0	91.1	90.6	1.0	95.6	0.93
**D_FF_Bp(16x16x16x16)**^a^	93.2	88.7	91.0	91.7	1.0	93.4	0.92
**D_FF_GNet(64)**^a^	90.7	90.2	90.5	90.6	1.0	94.0	0.90
**D_FF_Sn(48)**^a^	91.7	88.9	90.3	90.6	1.0	93.2	0.92
**D_FF_Bm(48)**^a^	91.6	88.9	90.2	90.6	1.0	93.2	0.91
**D_FF_Conic(48)**^a^	91.6	88.7	90.2	90.6	0.0	93.3	0.92
**D_FF_Bp(0)**^a^	89.1	90.3	89.7	89.6	2.1	93.8	0.91
**MLP**^b^	81.0	84.8	82.9	82.3	3.1	89.8	0.90
**RandomForest**^b^	86.6	65.3	75.9	78.7	1.6	80.6	0.86
**NaiveBayes**^b^	85.8	64.5	75.1	78.1	4.2	81.0	0.83
**RotationForest**^b^	88.3	60.8	74.6	78.1	0.0	79.1	0.85
**Logistic**^b^	80.7	67.9	74.3	76.0	1.0	80.8	0.63
**LogitBoost**^b^	81.0	61.2	71.1	73.4	1.6	77.6	0.81
**J48**^b^	77.4	60.7	69.0	71. 4	0.5	77.0	0.57
**SMO**^b^	96.8	27.9	62.4	70.3	9.9	61.5	0.65
**kNN**^b^	75.9	36.5	56.2	60.9	2.6	66.6	0.56

Employed machine learning systems are listed in decreasing order of overall accuracy. The results are the average of two testing experiments with training-testing A-B and B-A sequence. A hundred cases were presented in subset A and ninety-nine cases in subset B. *Overall accuracy* Arithmetic average of sensitivity and specificity, *Balanced accuracy* Weighted average of sensitivity and specificity, *PPV* Positive Predictive Value, *AUROC* Area Under the Receiver Operator Curve. Sensitivity, Specificity, Overall accuracy, Balanced accuracy, Variance and PPV are all expressed as percentage.

The best machine learning system reached ad accuracy of 91.4% with 94.1% sensitivity (correct prediction of positivity to SARS-COV-2) and 88.7% specificity (correct prediction of negativity to SARS-COV-2).

Table 7 shows the results obtained through applying a selected number of machine learning systems to the variables selected by the TWIST system. These results are the average of five Training-Testing procedures of a K-fold cross-validation protocol. Detailed predictive results for each experiment are available in Table S2 in the Online Supplement.

**Table 7:** Predictive results with 5-K fold protocol, using Semeion (^a^) and WEKA (^b^) machine learning systems

Machine learning system	Sensitivity	Specificity	Overall accuracy	Balanced accuracy	Variance	PPV	AUROC
**D_FF_Conic(6x12x12x12)**^a^	89.2	86.2	87.7	88.0	2.6	92.1	0.86
**D_FF_Conic(4x12x12x12)**^a^	87.5	84.6	86.0	86.5	3.4	91.0	0.84
**D_FF_Sn(48)**^a^	88.3	83.2	85.8	86.4	4.3	90.4	0.85
**D_FF_Bp(24)** ^a^	88.3	77.6	83.0	84.3	6.0	87.1	0.81
**RandomForest**^b^	90.0	58.0	74.0	78.1	3.2	78.9	0.83
**Logistic**^b^	82.5	62.5	72.5	75.1	5.6	79.7	0.74

Employed machine learning systems are listed in decreasing order of overall accuracy. The results are the average of five testing experiments. *Overall accuracy* Arithmetic average of sensitivity and specificity, *Balanced accuracy* Weighted average of sensitivity and specificity, *PPV* Positive Predictive Value, *AUROC* Area Under the Receiver Operator Curve. Sensitivity, Specificity, Overall accuracy, Balanced accuracy, Variance and PPV are all expressed as percentage.

## Discussion

In our exploratory, model development study, we analyzed data of 199 adult patients admitted to the emergency department of the largest hospital in Milan, Lombardy, with symptoms compatible with COVID-19 during the first 3 weeks of SARS-COV-2 outbreak in Italy. In the present manuscript, we describe this population and highlight the differences between patients who actually tested positive to SARS-COV-2 and those who did not. Few attempts in applying artificial intelligence to rapidly predict positivity/negativity to SARS-COV-2 were made since the outbreak, using mostly CT imaging and lab results, collected in Chinese population [35, 47, 48]. Nevertheless, we present the first European attempt and promising results applying artificial intelligence to predict the results of RT-PCR for SARS-COV-2, using only basic clinical data, available in the vast majority of emergency departments all over the world. The wide application of a similar decision support tool could have a major clinical and organizational impact during the current pandemic.

In our study, several differences were observed between the two study groups (Table 4). However, none of these, or a combination of them, allows, so far, to clearly differentiate between patients with COVID-19 and patients with other diseases, having a similar clinical presentation. Our data underline the key finding of Coronavirus-induced alterations in the white blood cell differential count (Table 4). On the one hand, in contrast to other reports [49–52] we did not observe a marked lymphocytopenia, possibly because of the early stage of the viral disease. On the other, other subtypes such as eosinophils might play a key role in COVID-19 [53].

When applying artificial intelligence to our dataset, in particular ANNs and MLS, we were able to predict with high sensitivity and specificity the results of RT-PCR for SARS-COV-2 (Table 6).

Artificial Neural Networks allow forecasting through understanding of the relationship between variables, in particular through the application of nonlinear relationships [40, 54, 55]. These systems initially learn from a set of data with a known solution (training). Thereafter, the networks, inspired by the analytical processes of the human brain, are able to reconstruct imprecise rules, which may be underlying a complex dataset (testing). Machine learning systems and, in particular, ANNs analyse real-world data very efficiently. The internal validity of their assessment is provided by uniquely strict validation protocols, seldom used in classical statistics [54, 56, 57].

In the present manuscript, it was possible to predict with reasonable accuracy the status of being positive or negative to SARS-COV-2 based on 42 simple variables. This was achieved using the TWIST algorithm, which does not have, at the moment, the same popularity of other techniques, such as K Fold, Boosting and others. Nevertheless, it has been used extensively in the past 15 years in different contexts [18, 58, 59]. The reason of its low diffusion is partly that TWIST is very complex to program, as it includes two evolutionary algorithms that work together managing a huge population of ANNs, kNN and Naive Bayes algorithms. The execution of TWIST needs therefore to be programmed in C language to be sufficiently fast. Thus, for its complexity and for needed running time, TWIST is not suitable for programming in Phyton, R or similar languages.

TWIST system allowed reaching a global accuracy of 91.4% with the best machine learning system: 94.1% sensitivity (correct prediction of positivity to SARS-COV-2) and 88.7% specificity (correct prediction of negativity to SARS-COV-2). Considering the eight best machine-learning systems their average performance was the following: sensitivity of 91.8%, specificity of 89.6% and global accuracy of 90.8%.

Comparing the two testing procedures (A-B and B-A), explained in the mathematical section, the differences in predicting values between these two experiment is small, therefore reasonably excluding overfitting of the model [16]. Furthermore, also the Cost Curves performed to assess mode calibration have shown acceptable results (Figs. S2-S5 of the Online Supplement).

In order to analyze our dataset also with a more popular and widely applied procedure, we applied a 5 k-fold cross-validation protocol, using a selected number of machine learning systems (Table 7). With this type of analysis, the best machine learning system obtained an overall accuracy of 87.7% with a sensibility and specificity of 89.2 and 86.2%, respectively. Global average performance was the following: sensitivity of 87.6%, specificity of 75.4% and a global accuracy of 81.5%.

Comparing these results with those obtained by the same machine learning systems, using the AB -BA Train-Testing protocol (shown in Table 6), the latter allows to obtain slightly better predictive results, reasonably related to the optimal splitting of the records, with an average performance of 89.1% sensibility, 82.2% specificity and 85.7% global accuracy. The high variance of results obtained with the K-Fold protocol and the low variance of the same results using TWIST protocol is suggestive of the high polarization affecting the K-Fold protocol with this kind of data. This is the reason why we have chosen to rely on an optimized distribution of records in training and testing subsets, rather than on a random allocation. Nevertheless, also the application of a standard K-fold cross-validation, i.e. a system widely available, was able to predict accurately the results of RT-PCR for SARS-COV-2.

It is useful to analyse variables selected by AI, as they certainly bear specific clinical information. As mentioned above, the white blood cells and their differential count are certainly very informative.

Indeed, total white blood cell count (R = − 0.46), lymphocytes (R = 0.26) and eosinophils (R = − 0.34) correlated either negatively or positively with the presence of COVID-19 and were included in the model.

In addition, the final model included also variables with very low correlation with RT-PCR results, such as dyspnea (R = − 0.07), basophils (R = -0.06), mean cell haemoglobin (R = − 0.06), non-invasive ventilation (R = − 0.05), monocytes (R = 0.05), age (R = 0.04), female sex (R = 0.02) and headache (R = 0.02). The fact that these variables have been included in the model confirms the ability of ANN to handle highly nonlinear functions.

Other authors have applied AI for the diagnosis of SARS-COV-2. Rao et al. employed an AI framework to a mobile phone-based survey, exclusively based on pre-hospital clinical symptoms and demographic characteristics to assess the probability of SARS-COV-2 infection [60]. Three different research groups tried to predict positivity to SARS-COV-2 using, among other variables, CT scans [14, 47, 61]. Chest CT scan was analysed via deep learning by Li et al. to differentiate SARS-COV-2 induced viral pneumonia from other lung diseases [62]. Two other research groups developed machine learning models and online applications, using only lab test results [35, 48].

Our model significantly differs from the abovementioned. First, it relies on basic clinical information, available in almost every emergency department. The required information is quickly obtainable for every patient at hospital admission. For this reason, we decided to include chest X-Ray rather than CT in our model. Indeed, despite CT being certainly more sensitive in identifying alterations typical of viral pneumonia [13], not every SARS-COV-2 suspect will have access to a CT scan. Second, our study is the first one analysing data from a European country. While there is no evidence so far, it is possible that different ethnicities will show slightly different responses to viral invasion.

### Limitations

This exploratory study has certainly several limitations. First, it is a retrospective, single-center study. Second, the study was conducted on a convenience sample of 200 patients. This aspect has two important implications: i) in this exploratory model development study no formal a priori sample size determination was performed and ii) the small sample size and the high number of input variables bear an intrinsic risk of over-fitting [37]. An additional limitation is the high exclusion rate (40%), potentially leading to a selection bias. However, the main reason for patients’ exclusion was the lack of data regarding the leukocyte formula. This exam was not part of the standard biochemical panel in our emergency department at the beginning of the pandemic. The clinical implications of this laboratory exam became quickly evident and the test was therefore frequently added to the biochemical panel later on in the pandemic. In light of this explanation and of the comparison between included and excluded patients (Table S3) we think that it is safe to exclude the presence of a selection bias. Furthermore, for organizational issues we were not able to perform an external validation, which certainly would have strengthened our results. In addition, some fundamental clinical data, such as arterial blood gas analysis were not available for all patients and this information was thus not included in the model. Given the typical profound hypoxemia of patients with COVID-19, it is conceivable that adding these variables to the system could further improve its accuracy. Finally, while no definitive data are available regarding the accuracy of RT-PCR testing for SARS-COV-2 [63], several studies have described a certain percentage of false negative results [6–8]. However, every negative result was re-tested after 48 h. This methodological aspect should reduce the risk of falsely negative results.

### Possible clinical and organizational implications

Facing a highly contagious viral outbreak requires a complex effort in terms of political, economic, social and health systems re-organization [64, 65]. A fundamental aspect is to define a clear management protocol, in order to separate infected from non-infected patients, i.e., those admitted for other clinical conditions. Indeed, it is of paramount importance to set up clearly separate pathways, in order to avoid the spread of viral infections within the hospital. A quick and reliable system to identify SARS-COV-2 infected patients is therefore fundamental. Currently, the gold standard for the diagnosis is a RT-PCR assay searching for SARS-COV-2 genome [9]. This type of molecular assay has certainly several limitations. During the first month of outbreak in Italy, the processing of samples became more efficient, theoretically reducing the technical time needed for the result. Despite this, the problem of delayed diagnosis still exists. This is due to the availability RT-PCR machines, considering the high demand during the outbreak. Furthermore, laboratories of referral hospitals, such as ours, analyse samples also arriving from smaller hospitals not equipped for SARS-COV-2 testing. Finally, of course, RT-PCR is not a perfect test, and false negative result have been described, even in the presence of strong clinical suspicion for COVID-19 [6–8].

For these reasons, applying AI as a rapid decision support tool for the diagnosis of COVID-19 and therefore to speed up the sorting of infected from non-infected patients would be of great clinical help. Indeed, a simple online software, fed with basic clinical data, easily obtainable in almost every emergency department, could apply trained ANNs to predict with high accuracy the RT-PCR result. The results obtained from this software should of course be integrated with available clinical data.

The application of AI to clinical practice is still limited for its complexity and for limited in-hospital availability of technical infrastructures and support. This, of course, could be particular troublesome for small centres with limited resources. The decision support software that could integrate the information contained in the present manuscript could ideally retrieve data directly from the electronic patient management system. Otherwise, data could be manually entered in an online software, which however significantly increases the risk of errors. An active support from and collaboration with the local information technology infrastructure is therefore fundamental in order to be able, in the future, to integrate AI into clinical practice.

Finally, it is conceivable that the information obtained from the present study might be useful also at the end of the current pandemic. Indeed, it is likely that SARS-COV-2 might become a seasonal virus. In this regard, the early identification would be a key factor to reduce the risk of a further epidemic outbreak.

## Conclusions

In summary, our exploratory study suggests that basic clinical data might be sufficient for properly trained ANNs and MLS algorithms to predict with good accuracy the positivity and negativity to SARS-COV-2. If confirmed in larger multicentre studies, this could have important clinical and organizational implications. Indeed, while not directly changing the treatment of COVID-19 patients, it could reduce the time patients spend unnecessarily in the emergency department awaiting the results of RT-PCR, could reduce the workload of intensive care staff and, finally, reduce the risk of collapsing healthcare systems.

## Supplementary Information

**Additional file 1.** Additional materials, methods and results.

## Data Availability

The dataset analysed during the current study is available from the corresponding author on reasonable request.

## Abbreviations

ANNs: Artificial Neural Networks
CT: Computed Tomography
COPD: Chronic obstructive pulmonary disease
COVID-19: Coronavirus Disease 2019
MLS: Machine Learning Systems
RT-PCR: Reverse Transcription- Polymerase Chain Reaction
SARS-COV-2: Severe Acute Respiratory Syndrome Coronavirus 2
TWIST: Training With Input Selection and Testing
