# Supplementary material for: Development of machine learning models to predict RT-PCR results for severe acute respiratory syndrome coronavirus 2 (SARS-CoV-2) in patients with influenza-like symptoms using only basic clinical data
Source: Scand J Trauma Resusc Emerg Med. 2020 Dec 1;28:113. doi: 10.1186/s13049-020-00808-8 (PMC7705856; doi:10.1186/s13049-020-00808-8)
Supplement: Supplementary file 1 — Additional file 1. Additional materials, methods and results. [file 13049_2020_808_MOESM1_ESM.docx]

**Development of Machine Learning models to predict RT-PCR results for Severe Acute Respiratory Syndrome Coronavirus 2 (SARS-CoV-2) in patients with influenza-like symptoms using only basic clinical data.**

**Online-only supplement**

Authored by Thomas Langer, Martina Favarato, Riccardo Giudici, Gabriele Bassi, Roberta Garberi, Fabiana Villa, Hedwige Gay, Anna Zeduri, Sara Bragagnolo, Alberto Molteni, Andrea Beretta, Matteo Corradin, Mauro Moreno, Chiara Vismara, Carlo Federico Perno, Massimo Buscema, Enzo Grossi, Roberto Fumagalli

**Corresponding Author**

Thomas Langer, MD; Department of Anesthesia and Intensive Care Medicine, Niguarda Ca’ Granda, Milan, Italy, Department of Medicine and Surgery, University of Milan-Bicocca, Monza, Italy; [thomas.langer@unimib.it](mailto:thomas.langer@unimib.it)

**Content of Online-only Supplement**

- Appendix 1: TWIST system
  - Figure S1: Synthetic representation of the dynamic of the TWIST algorithm
- Appendix 2: Model of calibration for AB-BA and 5-K fold protocols
  - Figure S2: ROC curves of the best performing Machine Learning models with half split train-test AB-BA
  - Figure S3: Cost curves of best performing Machine Learning Models with half split train-test AB-BA
  - Figure S4: ROC calibration curves of best performing Machine Learning Models with 5 K fold validation protocol.
  - Figure S5: Cost curves of the 4 best performing Machine Learning models with 5 K fold validation protocol
- Table S1: Extended predictive results for each machine learning, employed in the study
- Table S2: Extended predictive results for each machine learning, employed in the 5 K-fold protocol cross-validation (Semeion* and WEKA**)
- Table S3: Characteristics of the subgroup with missing leukocyte formula information and the study population

**Appendix 1: TWIST system**

When dealing with pattern recognition using machine learning systems, the kind of validation protocol and Inputs selection represent the most relevant problems. These problems can be expressed in this way:

1. How to generate an optimal couple of training and testing which is statistically representative of the assigned problem;
2. How to select the minimum number of input features able to maximize the accuracy of the dependent variables (target), in a blind test.

The TWIST algorithm (Training With Input Selection and Testing) is a possible scientific way to approach these two problems.

1. **Optimal distribution of records in training and testing subsets**

Any single distribution strategy of the source dataset in a training and in a testing and/or a validation set, (*i.e.* K Fold Cross Validation, Boosting, 5x2 Cross Validation, Training and Testing set splitting) is always executed with a random splitting of any single record (observation) of the source dataset.

The reason for the random criterion is of statistical nature. As the source dataset represents all the knowledge that we have of an assigned problem, we need to generate two subsets of data, which are more or less equivalent from a statistical point of view. Consequently, the training session will represent a good learning set for the machine learning, and the results of the testing session will be representative of the machine learning capability to generalize for the whole dataset. This criterion is valid, but it is not the only one.

In fact, the random criterion aims to optimize the following cost function:

(1) *f_1_*($d_{\Gamma}^{[tr]}$)*f_2_* ($d_{\Gamma}^{[ts]}$)*f_0_* ($d_{\Gamma}^{[global]}$);

where *f_1_*($d_{\Gamma}^{[tr]}$) and *f_2_* ($d_{\Gamma}^{[ts]}$) = probability density function of testing and training subset, respectively; *f_0_* ($d_{\Gamma}^{[global]}$) = probability density function of the global dataset.

This means that the random criterion aims to generate two subsets with, more or less, the same probability density function, and subset should be statistically equivalent to the global dataset.

The random criterion tries to approximate the cost function defined in equation (1). However, to optimize this cost function, we should consider every possible combination of each record into the two subsets and then, for any combination, measure and compare the probability density function of each subset. There is no evidence that the random criterion can optimize this cost function.

Therefore, given a dataset *D* _Γ_ of N records, the number of samples *d_Γ_*, which are comprised of K possible records, is given by

$\left(\begin{matrix} N \\ K \end{matrix}\right)$

Varying K, you have:

(2) $\sum_{K=0}^{N} \left(\begin{matrix} N \\ K \end{matrix}\right)=2^{N}$ (*)

(*) the search space is 2^N^, but the acceptable space is $\frac{2^{N}-2}{2}$.

Therefore, a pair of training and testing sets represents, on the solutions space, a possible solution

$x=\left(D_{\Gamma}^{[tr]},D_{\Gamma}^{[ts]}\right)$, given by the vector:

(3)

To optimize the cost function presented in equation (1), we propose an evolutionary algorithm whose population expresses, after each generation, different hypotheses about the splitting of the global dataset in two subsets.

Indeed, at any generation, each individual of the genetic population proposes which records of the global dataset have to be clustered into the subset A and which one into the subset B. From a technical viewpoint this is very easy: each individual of the genetic population is a vector of N Boolean values (1 or 0), where N is the number of the records of the global dataset.

The main problem, at this point, is to define a suitable fitness function, able to evaluate the validity of each hypothesis of the genetic population according to the equation (1): which hypothesis of splitting generates two subsets whose probability density functions are more similar?

To optimize these constraints we have used two independent Supervised Neural Networks (SNNs). Usually, we use a Multilayer Perceptron (Back Propagation based). The fitness evaluation of each hypothesis works in five independent steps, during which each individual of the genetic population presents its hypothesis of splitting the global dataset into two subsets, subset A and subset B:

1. The first SNN (SNN_A) is initialized and trained using the subset A, and it is stopped when the training error (that is, for example, the Root Mean Square Error or RMSE) is minimized;
2. The SNN_A, with the trained weights fixed, is applied in a blind way on the subset B, and its accuracy is saved;
3. The SNN_B, which is completely independent from the SNN_A, is initialized and trained using the subset B, and it is stopped when the training error (that is, for example, the RMSE) is minimized;
4. The SNN_B, with the trained weights fixed, is applied in a blind way on the subset A, and its accuracy is saved;
5. The minimum value of the two accuracies is assigned as fitness of the hypothesis of splitting, generated by any individual of the genetic population.

The steps from 1 to 5, named “Fitness Evaluation”, are executed for each individual of the genetic population, at any generation of the evolutionary algorithm.

The flow chart of the whole algorithm is the follow:

1. Genetic Population Initialization
2. Evolutionary Loop
   1. Fitness Evaluation of the hypothesis of splitting of each individual of the genetic population at the generation (n) (From step 1 to step 5);
   2. Crossover and offspring are produced;
   3. Random mutation is applied;
   4. Setup of the new population;
   5. If the average fitness continue to grow up start from the beginning; else terminate;
3. Save the subset A and the subset B with the best fitness.

This algorithm is named Training & Testing Optimization (for short T&T).

1. **How to select the minimum number of input features able to maximize the accuracy of the dependent variable prediction**

Feature selection techniques can be developed using two different general approaches, based on whether the selection of the variables is carried out dependently or independently of the learning algorithm used to build the inductor. The filter approach attempts to select the best attribute subset by evaluating its relevance based on the data. The “wrapper” approach, instead, requires that the selection of the best attribute subset considers as relevant those attributes that allow the induction algorithm to generate a more accurate performance.

Our Input Selection algorithm (I.S.) operates as a specific evolutionary wrapper system that responds to the need to reduce the dimensionality of the data by extracting the minimum number of variables necessary to control the “peaking” phenomenon and, at the same time, conserving the most information available.

IS is an evolutionary system conceived by M. Buscema in 2000, which is based on the GenD evolutionary algorithm. It is able to weigh the different variables of the available dataset, rather than burdening the inducer to learn which of them are relevant (1).

It operates as a specific evolutionary wrapper system for feature subset extraction. Unlike filter systems, that selecti features on the basis of data measures alone, IS uses the same learning algorithm for feature selection and evaluation (2).

The IS optimized sets are built considering only a subset of variables. Given a dataset $D_{\Gamma}$ of *N* records and *M* variables, which are divided into a training set $D_{\Gamma}^{[tr]}$ and a testing set $D_{\Gamma}^{[ts]}$, there are $\left(\begin{matrix} M \\ H \end{matrix}\right)$ possible samples $D'_{\Gamma}$, and then correspondingly $D'_{\Gamma}^{[tr]}$and $D'_{\Gamma}^{[ts]}$, of *H* extracted variables in which, varying *H*, it is also$\sum_{H=0}^{M} \left(\begin{matrix} M \\ H \end{matrix}\right)=2^{M}$. An inducer $\Omega_{D_{\Gamma}^{'\text{ }[tr]},A,F,Z}\left(\cdot\right)$ can be built with each sample, choosing an induction algorithm $A$, with its configuration parameters $F$and its initialization parameters $Z$.

It is possible to choose variables to achieve the best performance, in a transposed mode with respect to T&T, for a given pair of training and testing sets. Varying the input variables, we can consider that the goodness of the obtained results for each of the classifiers/predictors, basically depends upon whether the chosen variables, selected together, are relevant or not. We cannot know, in principle, the exact causes of the variable's relevance or irrelevance. The variables can be considered to be irrelevant by the system because they manifest redundant information, already present in other variables, or because they have no information at all, or because they manifest confusing information or noise. In any case, we have no reason to include these variables, when we can effectively eliminate them. As in T&T, the operation of analyzing the variables of the dataset before building the inducer constitutes another type of data mining, which also finalizes the extraction of the maximum amount of information present in the variables.

From a more technical perspective, IS is achieved with a GenD algorithm, considering, as space of solutions, the $\sum_{H=0}^{M} \left(\begin{matrix} M \\ H \end{matrix}\right)=2^{M}$ possible combinations of variables and using the following two-symbol alphabet:

$\Phi_{IS}=\left\{\text{ }\phi_{rel},\phi_{irrel}\right\}$

Where the first symbol ($\phi_{rel}$) represents a variable's belonging to the set of relevant variables $V_{rel}$, and the second symbol ($\phi_{irrel}$) represents a variable's belonging to the set of irrelevant variables $V_{irrel}$. A single possible solution $x=\left(V_{rel},V_{irrel}\right)$ is then a pair of relevant and irrelevant variable sets and is given by the vector:

$x=\left(V_{rel},V_{irrel}\right)=\left[x_{1},x_{2},\cdots,x_{M}\right]\in\begin{matrix} \begin{matrix} \Phi_{IS}^{M} & & & x_{i}\in\Phi_{IS} \end{matrix} & \end{matrix}$

In a similar way to the T&T, a *preliminary phase* and a *computational phase* are used to evaluate the parameters of the fitness function and, respectively, to extract the most relevant training and testing set variables. A standard back propagation artificial neural network is used as inducer $\Omega_{D_{\Gamma}^{[tr]},A,F,Z}\left(\cdot\right)$ for the fitness function. It is configured during the preliminary phase and used during the successive computational phase with both fixed configuration and initialization parameters (weights).

In the preliminary phase, particular attention is given to configuring the ANN parameters in order to avoid potential overfitting problems, which can arise when only a small amount of data is available with a large number of variables. (3, 4) The number $E_{0}$ of epochs used to train the inducer is carefully evaluated so as to stop its learning phase on the training data before overfitting occurs.

GenD applied to IS usefully proposes other choices in addition to selecting the best set of variables, with a fitness less or equal to the best. These can be chosen as a practical library of input selections to be used when the best choice of variables is not accessible or is not convenient due to economic or other reasons.

As is typical for genetic wrapper systems, compared to other machine learning approaches, IS can exhibit a heavier computational time, but it also presents some useful advantages. When compared to decision tree algorithms, such as ID3, C4.5 and CART or to instance-based algorithms, like IBL, IS presents a greater performance robustness to the presence of many irrelevant features. Whereas, in comparison with naive-Bayes algorithms, a robustness to a presence of correlated features, even if relevant, can be observed. IS inherits the robustness of gradient based neural networks, like back propagation (4), and the flexibility of evolutionary algorithms to explore the space of the program solutions.

To integrate our IS algorithm with T&T algorithm in only one procedure, we have to modify the structure of each individual of the genetic population, that we have already described. This fusion will generate a complex algorithm able to look for the best distribution of the global sample into two optimal subsets with the minimum of input features, useful for optimal pattern recognition. We have named this new algorithm TWIST (Training With Input Selection and Testing).

This integration is easy: in T&T algorithm each individual of the genetic population is a vector of N component of Boolean values, where N is the number of the records of the global dataset: when the value of a generic component of the vector is 1, then that record is saved into the subset A, and when the value is 0, then the record is saved into the subset B.

In I.S. algorithm, instead, each individual of the genetic population has to be a vector of M component of Boolean values, where M is the number of all the input variables of the global dataset: in this case, when the value of a generic component of the vector is 1 the corresponding input feature is saved into the subset A and the subset B, while if the value is 0, then the corresponding input feature is removed.

Consequently, in TWIST algorithm every individual of the genetic population will be defined by two vectors of different length:

1. The first one, showing its hypothesis about which records (*N*) has to be stored into the subset A and which ones have to be stored into the subset B;
2. The second one, showing its hypothesis about which inputs (*M*) have to be used into the two subsets and which one have to be deleted.

After this modification the TWIST algorithm works as well as the T&T algorithm, already described.

At the end of its evolution, TWIST will generate two subsets of data with a very similar probability density of distribution and with the minimal number of effective variables for pattern recognition.

**Figure S1. Synthetic representation of the dynamic of the TWIST algorithm.**


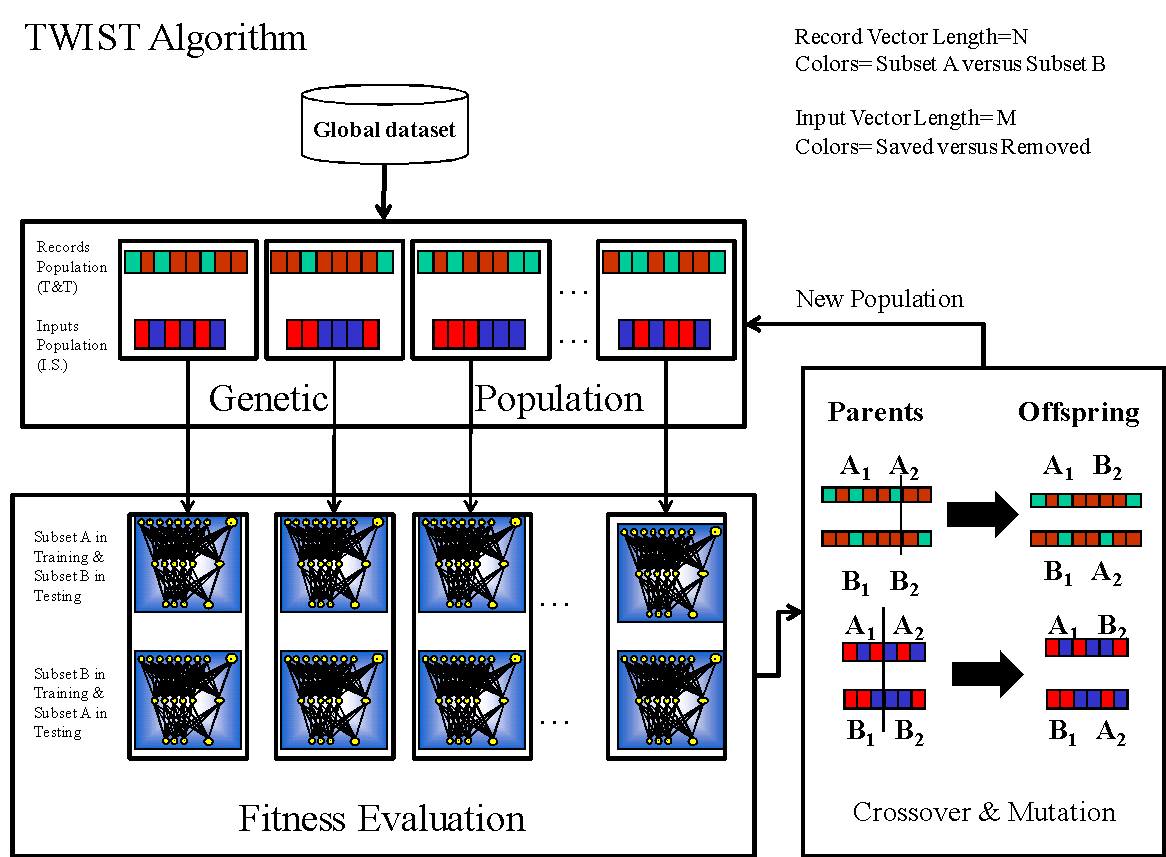


**Appendix 2: ROC and Cost Curves for AB-BA and 5-K fold protocols**

**Figure S2**: ROC curves of the best performing Machine Learning models with half split train-test AB-BA


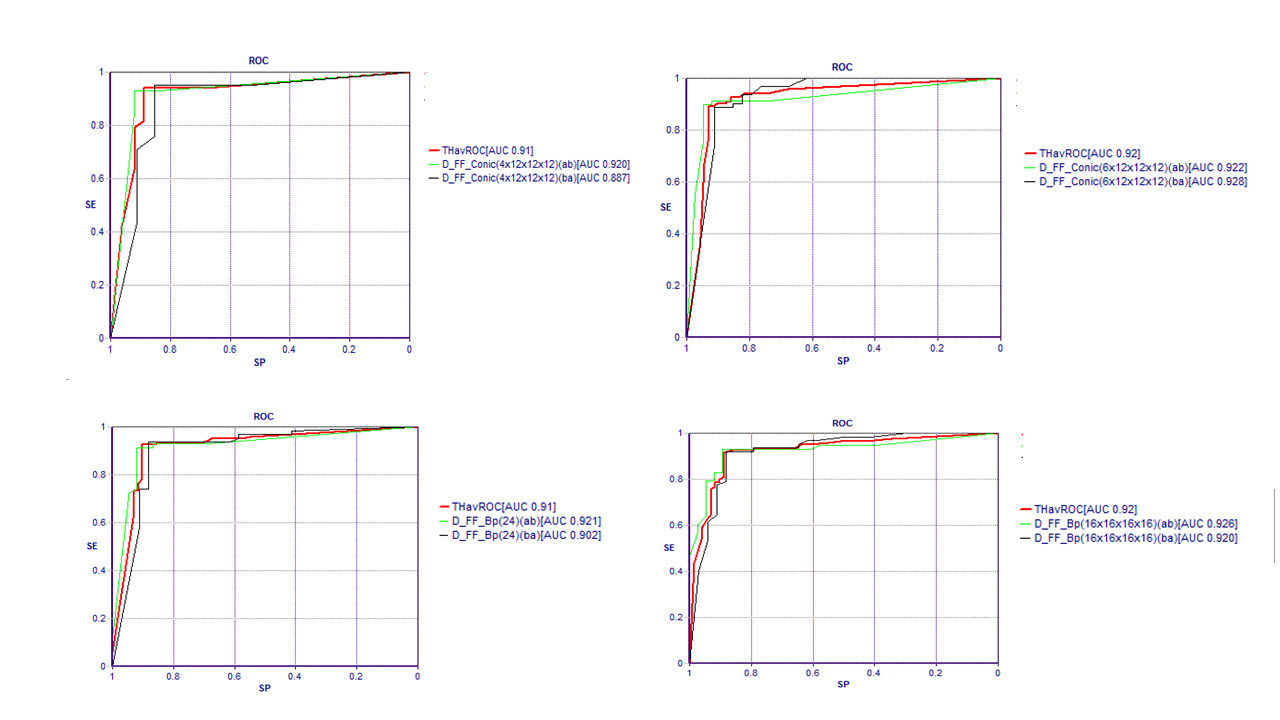


**Figure S3:** Cost curves of best performing Machine Learning Models with half split train-test AB-BA


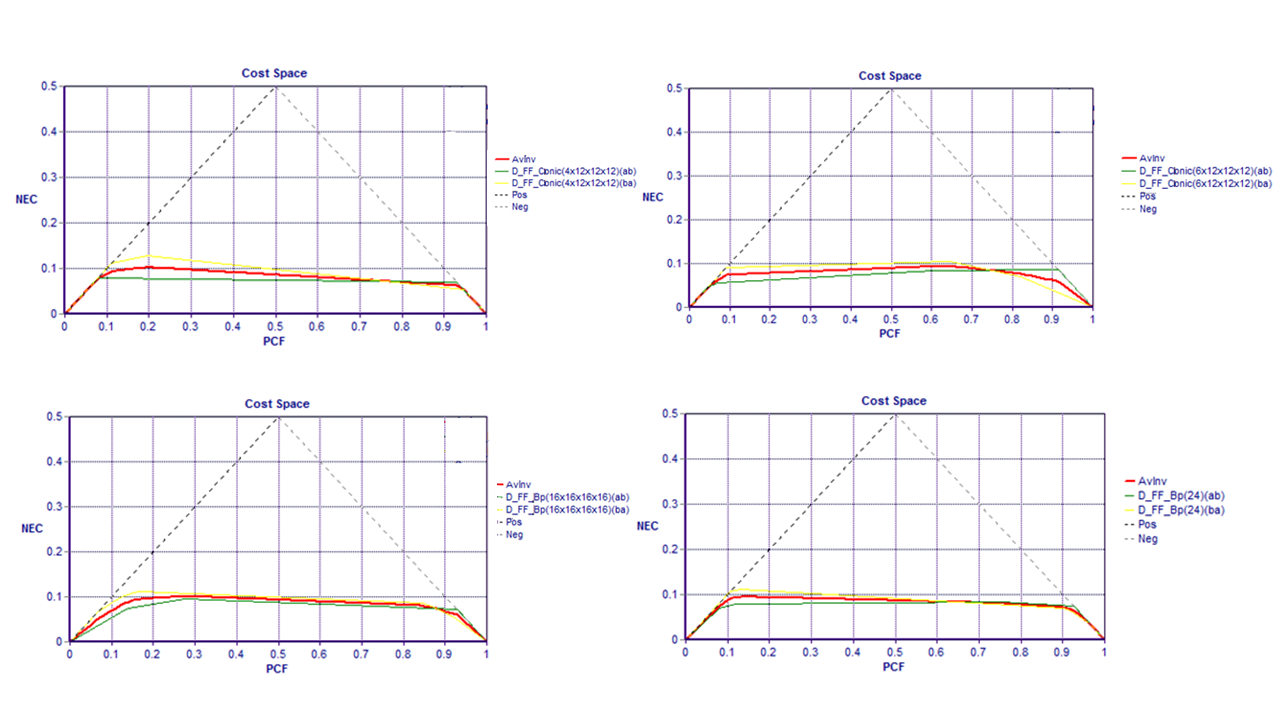


NEC = Normalized Expected Cost; PCF =Probability Cost Function.

**Figure S4:** ROC calibration curves of best performing Machine Learning Models with 5 K fold validation protocol.


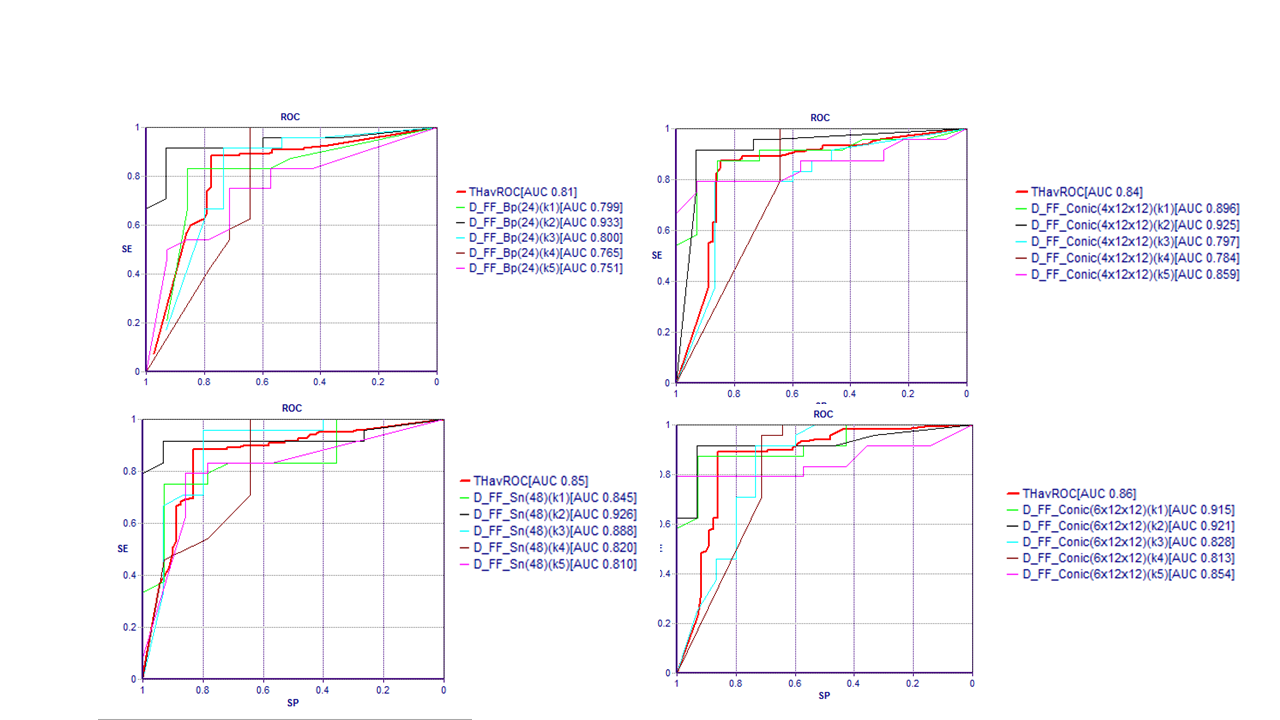


**Figure S5:** Cost curves of the 4 best performing Machine Learning models with 5 K fold validation protocol


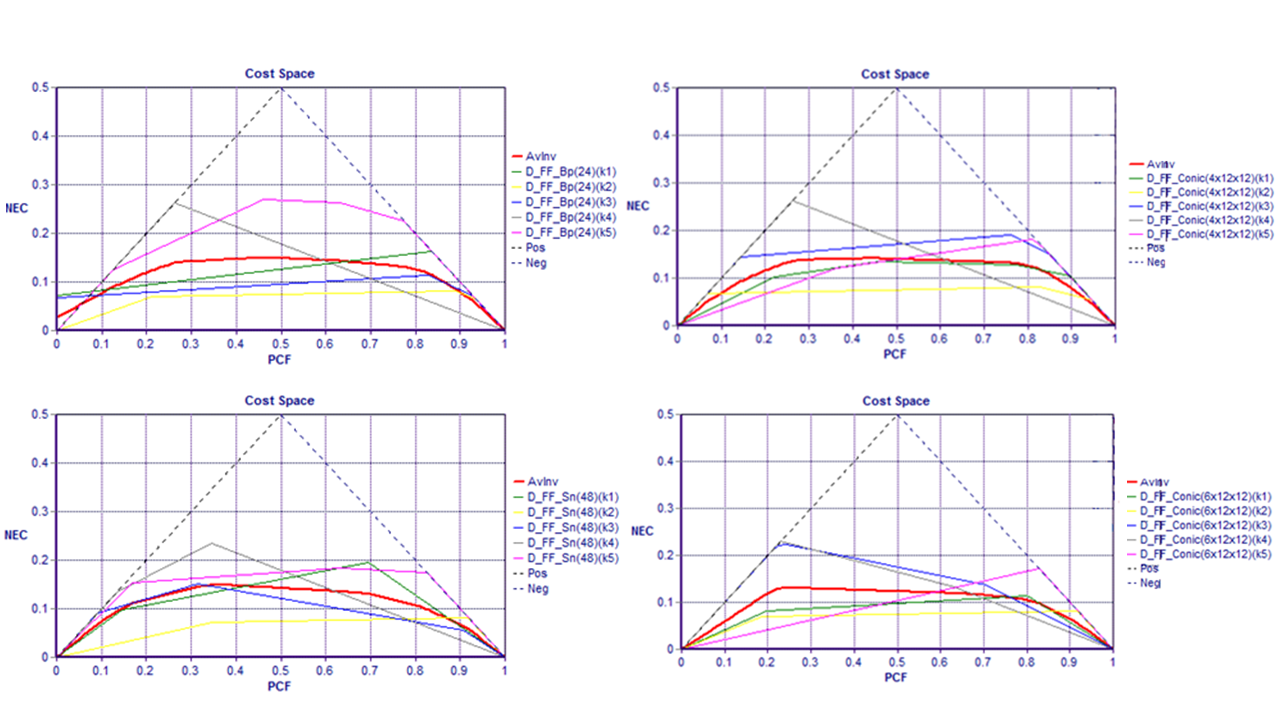


NEC = Normalized Expected Cost; PCF =Probability Cost Function.

**Table S1. Extended predictive results for each machine learning, employed in the study (Semeion* and WEKA**).**

| **Machine learning system** | **Sensitivity (%)** | **Specificity (%)** | **Overall accuracy^1^ (%)** | **Balanced accuracy^2^ (%)** | **PVV^3^ (%)** | **AUROC^4^** |
| --- | --- | --- | --- | --- | --- | --- |
| **D_FF_Conic(4x12x12x12)*** | | | | | | |
| A-B | 93.1 | 92.1 | 92.6 | 92.7 | 94.7 | 0.92 |
| B-A | 95.2 | 85.3 | 90.2 | 91.7 | 92.2 | 0.89 |
| **Mean** | **94.1** | **88.7** | **91.4** | **92.2** | **93.5** | **0.90** |
| **D_FF_Bp(24)*** | | | | | | |
| A-B | 89.7 | 94.7 | 92.2 | 91.7 | 96.3 | 0.92 |
| B-A | 88.7 | 91.2 | 89.9 | 89.6 | 94.8 | 0.93 |
| **Mean** | **89.2** | **93.0** | **91.1** | **90.6** | **95.6** | **0.93** |
| **D_FF_Conic(6x12x12x12)*** | | | | | | |
| A-B | 91.4 | 92.1 | 91.7 | 91.7 | 94.6 | 0.92 |
| B-A | 93.6 | 88.2 | 90.9 | 91.7 | 93.6 | 0.90 |
| **Mean** | **92.5** | **90.2** | **91.3** | **91.7** | **94.1** | **0.91** |
| **D_FF_Sn(48)*** | | | | | | |
| A-B | 93.1 | 89.5 | 91.3 | 91.7 | 93.1 | 0.93 |
| B-A | 90.3 | 88.2 | 89.3 | 89.6 | 93.3 | 0.92 |
| **Mean** | **91.7** | **88.9** | **90.3** | **90.6** | **93.2** | **0.92** |
| **D_FF_Conic(48)*** | | | | | | |
| A-B | 89.7 | 92.1 | 90.9 | 90.6 | 94.6 | 0.93 |
| B-A | 93.6 | 85.3 | 89.4 | 90.6 | 92.1 | 0.91 |
| **Mean** | **91.6** | **88.7** | **90.2** | **90.6** | **93.3** | **0.92** |
| **D_FF_Bp(16x16x16x16)*** | | | | | | |
| A-B | 89.7 | 92.1 | 90.9 | 90.6 | 94.6 | 0.92 |
| B-A | 96.8 | 85.3 | 91.0 | 92.7 | 92.3 | 0.92 |
| **Mean** | **93.2** | **88.7** | **91.0** | **91.7** | **93.4** | **0.92** |
| **D_FF_GNet(64)*** | | | | | | |
| A-B | 87.9 | 92.1 | 90.0 | 89.6 | 94.4 | 0.90 |
| B-A | 93.6 | 88.2 | 90.9 | 91.7 | 93.6 | 0.90 |
| **Mean** | **90.7** | **90.2** | **90.5** | **90.6** | **94.0** | **0.90** |
| **D_FF_Bm(48)*** | | | | | | |
| A-B | 89.7 | 89.5 | 89.6 | 89.6 | 92.9 | 0.92 |
| B-A | 93.6 | 88.2 | 90.9 | 91.7 | 93.6 | 0.91 |
| **Mean** | **91.6** | **88.9** | **90.2** | **90.6** | **93.2** | **0.91** |
| **D_FF_Bp(0)*** | | | | | | |
| A-B | 86.2 | 89.5 | 87.8 | 87.5 | 92.6 | 0.92 |
| B-A | 91.9 | 91.2 | 91.6 | 91.7 | 95.0 | 0.90 |
| **Mean** | **89.1** | **90.3** | **89.7** | **89.6** | **93.8** | **0.91** |
| **MLP**** | | | | | | |
| A-B | 86.2 | 84.2 | 85.2 | 85.4 | 89.3 | 0.88 |
| B-A | 75.8 | 85.3 | 80.6 | 79.2 | 90.4 | 0.86 |
| **Mean** | **81.0** | **84.8** | **82.9** | **82.3** | **89.8** | **0.87** |
| **RandomForest**** | | | | | | |
| A-B | 84.5 | 65.8 | 75.1 | 77.1 | 79.0 | 0.86 |
| B-A | 88.7 | 64.7 | 76.7 | 80.2 | 82.1 | 0.87 |
| **Mean** | **86.6** | **65.6** | **75.9** | **78.7** | **80.6** | **0.86** |
| **NaiveBayes**** | | | | | | |
| A-B | 84.5 | 79.0 | 81.7 | 82.3 | 86.0 | 0.84 |
| B-A | 87.1 | 50.0 | 68.6 | 74.0 | 76.1 | 0.83 |
| **Mean** | **85.8** | **64.5** | **75.1** | **78.1** | **81.0** | **0.83** |
| **RotationForest**** | | | | | | |
| A-B | 86.2 | 65.8 | 76.0 | 78.1 | 79.4 | 0.87 |
| B-A | 90.3 | 55.9 | 73.1 | 78.1 | 78.9 | 0.82 |
| **Mean** | **88.3** | **60.8** | **74.6** | **78.1** | **79.1** | **0.85** |
| **Logistic**** | | | | | | |
| A-B | 77.6 | 71.1 | 74.3 | 75.0 | 80.4 | 0.63 |
| B-A | 83.9 | 64.7 | 74.3 | 77.1 | 81.3 | 0.63 |
| **Mean** | **80.7** | **67.9** | **74.31** | **76.0** | **80.8** | **0.63** |
| **LogitBoost**** | | | | | | |
| A-B | 84.5 | 60.5 | 72.5 | 75.0 | 76.6 | 0.83 |
| B-A | 77.4 | 61.8 | 69.6 | 71.9 | 78.7 | 0.78 |
| **Mean** | **81.0** | **61.2** | **71.1** | **73.4** | **77.6** | **0.81** |
| **J48**** | | | | | | |
| A-B | 74.1 | 68.4 | 71.3 | 71.9 | 78.2 | 0.67 |
| B-A | 80.7 | 52.9 | 66.8 | 70.8 | 75.8 | 0.47 |
| **Mean** | **77.4** | **60.7** | **69.0** | **71.4** | **77.0** | **0.57** |
| **SMO**** | | | | | | |
| A-B | 100.0 | 0.0 | 50.0 | 60.4 | 60.4 | 0.58 |
| B-A | 93.6 | 55.8 | 74.8 | 62.6 | 62.6 | 0.72 |
| **Mean** | **96.8** | **27.9** | **62.4** | **61.5** | **61.5** | **0.65** |
| **kNN**** | | | | | | |
| A-B | 77.6 | 29.0 | 53.3 | 58.3 | 62.5 | 0.53 |
| B-A | 74.2 | 44.1 | 59.2 | 63.5 | 70.8 | 0.59 |
| **Mean** | **75.9** | **36.5** | **56.2** | **60.9** | **66.6** | **0.56** |
| *This table lists the results for both testing experiment (Training Set A- Testing Set B and Training Set B- Testing Set A).*  **^1^**Aritmetic mean of sensitivity and specificity  **^2^** Weighted mean of sensitivity and specificity  **^3^**PPV-Positive Predictive Value.  **^4^**AUROC- Area Under the Receiver Operator Curve. | | | | | | |

**Table S2. Extended predictive results for each machine learning, employed in the 5 K-fold protocol cross-validation (Semeion* and WEKA**).**

| **Machine learning system 5 K-fold experiments** | **Sensitivity (%)** | **Specificity(%)** | **Overall accuracy(%)^1^** | **Balance accuracy(%)^2^** | **PVV(%)^3^** | **AUROC^4^** |
| --- | --- | --- | --- | --- | --- | --- |
| **D_FF_Bp(24)*** | | | | | | |
| k1 | 83.3 | 85.7 | 84.5 | 84.2 | 90.9 | 0.80 |
| k2 | 91.7 | 93.3 | 92.5 | 92.3 | 95.7 | 0.93 |
| k3 | 91.7 | 73.3 | 82.5 | 84.6 | 84.6 | 0.80 |
| k4 | 100.0 | 64.3 | 82.1 | 86.8 | 82.86 | 0.77 |
| k5 | 75.0 | 71.4 | 73.2 | 73.7 | 81.8 | 0.75 |
| **Mean** | **88.3** | **77.6** | **83.0** | **84.3** | **87.3** | **0.81** |
| **D_FF_Conic(4x12x12)*** | | | | | | |
| k1 | 87.5 | 85.7 | 86.6 | 86.8 | 91.3 | 0.90 |
| k2 | 91.7 | 93.3 | 92.5 | 92.3 | 95.7 | 0.93 |
| k3 | 79.2 | 86.7 | 82.9 | 82.1 | 90.5 | 0.80 |
| k4 | 100.0 | 64.3 | 82.1 | 86.8 | 82.8 | 0.78 |
| k5 | 79.2 | 92.9 | 86.0 | 84.2 | 95.0 | 0.86 |
| **Mean** | **87.5** | **84.6** | **86.0** | **86.5** | **91.0** | **0.84** |
| **D_FF_Sn(48)*** | | | | | | |
| k1 | 75.0 | 92.9 | 83.9 | 81.6 | 94.7 | 0.85 |
| k2 | 91.7 | 93.3 | 92.5 | 92.3 | 95.7 | 0.93 |
| k3 | 95.8 | 80.0 | 87.9 | 89.7 | 88.5 | 0.89 |
| k4 | 100.0 | 64.3 | 82.1 | 86.8 | 82.8 | 0.82 |
| k5 | 79.2 | 85.7 | 82.4 | 81.6 | 90.5 | 0.81 |
| **Mean** | **88.3** | **83.2** | **85.8** | **86.4** | **90.4** | **0.85** |
| **D_FF_Conic(6x12x12)*** | | | | | | |
| k1 | 87.5 | 92.9 | 90.2 | 89.5 | 95.5 | 0.92 |
| k2 | 91.7 | 93.3 | 92.5 | 92.3 | 95.7 | 0.92 |
| k3 | 91.7 | 73.3 | 82.5 | 84.6 | 84.6 | 0.83 |
| k4 | 95.8 | 71.4 | 83.6 | 86.8 | 85.2 | 0.81 |
| k5 | 79.2 | 100.0 | 89.6 | 86.8 | 100.0 | 0.85 |
| **Mean** | **89.2** | **86.2** | **87.7** | **88.0** | **92.2** | **0.86** |
| **Logistic**** | | | | | | |
| k1 | 87.5 | 64.3 | 75.9 | 79.0 | 80.8 | 0.81 |
| k2 | 70.8 | 86.7 | 78.8 | 76.9 | 89.5 | 0.83 |
| k3 | 79.2 | 40.0 | 59.6 | 64.1 | 67.9 | 0.64 |
| k4 | 100.0 | 42.9 | 71.4 | 79.0 | 75.0 | 0.50 |
| k5 | 75.0 | 78.6 | 76.8 | 76.3 | 85.7 | 0.85 |
| **Mean** | **82.5** | **62.5** | **72.5** | **75.1** | **79.8** | **0.74** |
| **RandomForest**** | | | | | | |
| k1 | 87.5 | 57.1 | 72.3 | 76.3 | 77.8 | 0.83 |
| k2 | 83.3 | 80.0 | 81.7 | 82.1 | 87.0 | 0.85 |
| k3 | 87.5 | 60.0 | 73.8 | 76.9 | 77.8 | 0.87 |
| k4 | 100.0 | 28.6 | 64.3 | 73.7 | 70.6 | 0.83 |
| k5 | 91.7 | 64.3 | 78.0 | 81.6 | 81.5 | 0.81 |
| **Mean** | **90.0** | **58.0** | **74.0** | **78.1** | **78.9** | **0.83** |
| *This table lists the results for each experiment. The resulting mean is the value reported in the main manuscript in Table 6.*  **^1^**Aritmetic mean of sensitivity and specificity  **^2^** Weighted mean of sensitivity and specificity  **^3^** PPV-Positive Predictive Value.  **^4^** AUROC- Area Under the Receiver Operator Curve. | | | | | | |

**Table S3. Characteristics of the subgroup with missing leukocyte formula information and the study population.**

| **Demographic characteristics** | **Study population (n=199)** | **Excluded patients (n=139)** | **p value** |
| --- | --- | --- | --- |
| Male ̶ no. (%) | 127 (63.8) | 78 (56.1) | 0.19 |
| Age, years | 65 [46-78] | 71 [51-82] | 0.08 |
| **Comorbidities ̶ no. (%)** | | | |
| Hypertension | 85 (42.7) | 57 (41.0) | 0.84 |
| Ischemic cardiomyopathy | 19 (9.6) | 20 (14.4) | 0.23 |
| Atrial fibrillation | 19 (9.6) | 13 (9.4) | 0.90 |
| Diabetes mellitus | 29 (14.6) | 27 (19.4) | 0.30 |
| Chronic kidney disease | 11 (5.5) | 11 (7.9) | 0.52 |
| Solid organ tumour | 16 (8.0) | 17 (12.2) | 0.28 |
| Hematologic malignancy | 6 (3.0) | 9 (6.5) | 0.21 |
| Human immunodeficiency virus | 1 (0.5) | 1 (0.7) | 0.64 |
| Chronic liver disease | 7 (3.5) | 4 (2.9) | 0.99 |
| Autoimmune/inflammatory disease | 6 (3.0) | 7 (5.0) | 0.51 |
| Chronic obstructive pulmonary disease (COPD) | 15 (7.5) | 15 (10.8) | 0.40 |
| Asthma | 13 (6.6) | 7 (5.0) | 0.73 |
| Chronic Interstitial lung disease | 1 (0.5) | 3 (2.2) | 0.38 |
| Smoking (active) | 12 (6.0) | 10 (7.2) | 0.84 |
| **Current medication ̶ no. (%)** | | | |
| Beta blockers | 40 (20.1) | 33 (23.8) | 0.51 |
| Calcium channel antagonists | 27 (13.6) | 17 (12.2) | 0.85 |
| Sartans | 24 (12.1) | 11 (7.9) | 0.29 |
| Diuretics | 35 (17.6) | 27 (19.4) | 0.68 |
| Angiotensin converting enzyme inhibitors | 33 (16.6) | 22 (15.8) | 0.97 |
| Hydroxymethylglutaryl-CoA (HMG-CoA) reductase inhibitors | 28 (14.1) | 29 (20.9) | 0.14 |
| Anti-arrhythmic drugs | 11 (5.5) | 8 (5.8) | 0.88 |
| Anticoagulant drugs | 25 (12.6) | 8 (5.8) | 0.06 |
| Antiplatelets drugs | 33 (16.6) | 33 (23.7) | 0.14 |
| Anti-epileptic drugs | 10 (5.0) | 5 (3.6) | 0.72 |
| Drugs for psychiatric disorders | 7 (3.5) | 8 (5.8) | 0.48 |
| Immunosuppressant drugs | 12 (6.0) | 15 (10.8) | 0.17 |
| 5-alpha-reductase inhibitors | 12 (6.0) | 15 (10.8) | 0.17 |
| Antibiotics before Emergency Department | 47 (23.6) | 41 (29.5) | 0.28 |
| **Symptoms ̶ no. (%)** | | | |
| Fever | 174 (87.4) | 111 (79.9) | 0.08 |
| Cough | 130 (65.3) | 77 (55.4) | 0.08 |
| Headache | 12 (6.0) | 2 (1.4) | 0.99 |
| Asthenia | 18 (9.1) | 10 (7.6) | 0.79 |
| Arthralgia | 7 (3.5) | 7 (5.0) | 0.68 |
| Sore throat | 8 (4.0) | 4 (2.8) | 0.80 |
| Gastrointestinal symptoms | 27 (13.6) | 17 (12.2) | 0.85 |
| Chest pain | 7 (3.5) | 8 (5.8) | 0.46 |
| Dyspnea | 75 (37.7) | 56 (40.3) | 0.64 |
| Syncope | 5 (2.5) | 2 (1.4) | 0.98 |
| **Clinical findings** | | | |
| Glasgow coma scale, score | 15 [15-15] | 15 [15-15] | 0.60 |
| Sinus rhythm ̶ no. (%) | 185 (93.0) | 127 (91.4) | 0.74 |
| Normal ST segment ̶ no. (%) | 198 (99.5) | 136 (97.8) | 0.38 |
| Heart rate, beats per min | 90 [80-105] | 90 [80- 109] | 0.93 |
| Respiratory rate, breaths per min | 18 [16-22] | 18 [16-22] | 0.62 |
| Systolic blood pressure, mmHg | 130 [118 -145] | 135 [120 -150] | 0.06 |
| Diastolic blood pressure, mmHg | 75 [65-80] | 70 [65-80] | 0.44 |
| Body temperature, Celsius | 37.6 [37.0 – 38.2] | 37.5 [36.5 – 38.4] | 0.37 |
| Oxygen supplementation (nasal cannula. mask) ̶ no. (%) | 51 (25.6) | 41 (29.7) | 0.48 |
| Non-Invasive Ventilation ̶ no. (%) | 8 (4.0) | 4 (3.0) | 0.84 |
| Chest X-rays opacity ̶ no. (%) | 158 (79.4) | 86 (64.2) | 0.003 |
| Chest X-rays pleural effusion ̶ no. (%) | 20 (10.1) | 28 (20.9) | 0.009 |
| Total white blood cell count, 10^3^/µL | 6.64 [4.65-9.65] | 7.23 [4.96-10.12] | 0.08 |
| Total red blood cells count, 10^6^/µL | 4.80 [4.29-5.25] | 4.63 [4.1-4.99] | 0.01 |
| Haemoglobin, g/dL | 13.8 [12.3 – 15] | 13.5 [11.7 – 14.5] | 0.001 |
| Haematocrit, % | 41.6 [18.4 – 45.3] | 40.6 [36.3 -43.6] | 0.01 |
| Mean red blood cell volume, µm^3^ | 88.3 [85.4-91.4] | 89.9 [85.0 - 92.7] | 0.13 |
| Mean corpuscular haemoglobin, pg | 29.2 [28.1-30.5] | 29.0 [27.7-30.5] | 0.49 |
| Mean corpuscular haemoglobin concentration, g/dL | 33.1 [32.0-33.9] | 32.5 [31.8-33.3] | <0.001 |
| Red blood cells distribution width,**%** | 13.2 [12.4-14.4] | 13.3 [12.4-14.6] | 0.32 |
| Platelets, 10^3^/µL | 193 [162-244] | 202 [160 – 256] | 0.18 |
| Creatinine, mg/dL | 1.0 [0.80-1.30] | 1.0 [0.81-1.28] | 0.67 |
| Glicemia, mg/dL | 120 [104-142] | 116 [103-146] | 0.72 |
| Urea, mg/dL | 35 [23-54.5] | 37 [27-55] | 0.50 |
| Calcium, mg/dL | 9.0 [8.7-9.4] | 9.0 [8.5-9.3] | 0.67 |
| Sodium, mEq/L | 138 [135-140] | 139 [136-141] | 0.01 |
| Total bilirubin, mg/dL | 0.5 [0.4-0.7] | 0.6 [0.4-0.8] | 0.07 |
| C-reactive protein, mg/dL | 4.1 [1.2-9.0] | 4.5 [1.1 – 8.9] | 0.96 |
| SARS-COV-2 Infected ̶ no. (%) | 124 (62.3) | 52 (37.4) | <0.001 |
| Variables are expressed as median [interquartile range], mean ± Standard Deviation or as number (percentages). Clinical findings refer to data gathered in the Emergency Room. | | | |

**REFERENCES**

1. Rumelhart DE. Parallel Distributed Processing: Explorations in the Microstructure of Cognition, Vol. 1: Foundations: MIT Press; 1986.

2. Kohavi R, John G. Wrappers for feature selection. Artificial Intelligence - AI. 1997;1.

3. Ak J, Zongker D. Feature Selection: Evaluation, Application, and Small Sample Performance. Pattern Analysis and Machine Intelligence, IEEE Transactions on. 1997;19:153-8.

4. Rumelhart DE, Chauvin Y, Ph. D. Back propagation : theory, architectures, and applications. Hillsdale, N.J: Lawrence Erlbaum Associates; 1995.
